# Supplementary material for: Correlations between social media addiction and anxiety, depression, FoMO, loneliness and self-esteem among students: A systematic review and meta-analysis
Source: PLoS One. 2025 Sep 24;20(9):e0329466. doi: 10.1371/journal.pone.0329466 (PMC12459768; doi:10.1371/journal.pone.0329466)
Supplement: S1 File — (DOCX) [file pone.0329466.s001.docx]

**Correlations between social media addiction and anxiety, depression, FOMO, loneliness and self-esteem among students: A systematic review and meta-analysis**

Zhang Jing^1^, Wang Yang^2^, Zhou Lei^1^, Wu Junmei^3^, Li Hui^4*^, Zhu Tianmin^1*^

1. School of Rehabilitation and Health Preservation, Chengdu University of Traditional Chinese Medicine, Chengdu, China
2. School of Sports Medicine and Health, Chengdu Sport University, Chengdu, China
3. School of Acupuncture and Tuina, Chengdu University of Traditional Chinese Medicine, Chengdu, China
4. School of Preclinical Medicine, Chengdu University, Chengdu, China

*Corresponding author

E-mail address: tmz13608216905@163.com; [ttlihui@163.com](mailto:ttlihui@163.com)

**ABSTRACT:**

***Background and aims:*** With the ubiquity of internet, social media become an essential part of the daily life. The social media includes various types, such as Facebook, Twitter, TikTok, WeChat, and so on. It has tight links with the psychological issues, self-esteem, Fear of Missing Out (FoMO), attachment and loneliness. In order to further explore the connections between SMA (social media addiction) and these factors, we thus performed a meta-analysis to quantitatively synthesize the previous findings.

***Methods:*** PubMed, Embase, Web of Science, Chinese National Knowledge Infrastructure (CNKI), Chinese Biological Medicine (CBM) and Technology Journal Database (VIP) databases have been accessed for a systematic review and meta-analysis. Pooled Pearson’s correlation coefficients between SMA and anxiety, depression, loneliness, FoMO and self-esteem were calculated by STATA software using random or fixed effects model.

***Results:*** Twenty-seven studies involving a total of 25719 students were identified. This meta-analysis indicated the positive correlations between social media addiction and anxiety, depression, loneliness and FoMO (anxiety: summary r = 0.31, 95% CI = 0.25–0.36, P < 0.001; depression: summary r = 0.31, 95% CI = 0.27–0.34, P < 0.001; loneliness: summary r = 0.21, 95% CI = 0.13–0.29, P < 0.00; FoMO: summary r = 0.41, 95% CI = 0.36–0.45, P < 0.001). Negative correlation was also found between self-esteem and SMA (self-esteem: summary r = -0.24，95% Cl = -0.26– -0.22, P<0.001).

***Conclusions:*** There were significant positive associations between SMA and anxiety, depression, loneliness, FoMO and negative associations between self-esteem and SMA. These factors interact with social media use and create more social media dependency, leads to the social media addiction finally. This meta-analysis can offer prevention for the social media overuse through observing the correlation between these factors and SMA. The viewpoints also can give the direction for correct interventions for the social media addiction.

**KEYWORDS:**

Social media addiction, self-esteem, loneliness, depression, FoMO, anxiety, meta-analysis

**Author list**

**Zhang Jing**

1. School of Rehabilitation and Health Preservation, Chengdu University of Traditional Chinese Medicine, Chengdu, China

E-mail: zhangjing156299@163.com

**Wang Yang**

2. School of Sports Medicine and Health, Chengdu Sport University, Chengdu, China

E-mail: wangyang@cdsu.edu.cn

**Zhou Lei**

1. School of Rehabilitation and Health Preservation, Chengdu University of Traditional Chinese Medicine, Chengdu, China

E-mail: [2429480502@qq.com](mailto:2429480502@qq.com)

**Wu Junmei**

3. School of Acupuncture and Tuina, Chengdu University of Traditional Chinese Medicine, Chengdu, China

E-mail: 1696065983@qq.com

**Li Hui***

4. School of Preclinical Medicine, Chengdu University, Chengdu, China

E-mail: [ttlihui@163.com](mailto:ttlihui@163.com)

**Zhu Tianmin***

1. School of Rehabilitation and Health Preservation, Chengdu University of Traditional Chinese Medicine, Chengdu, China

E-mail: tmz13608216905@163.com
