# Supplementary material for: Correlations between social media addiction and anxiety, depression, FoMO, loneliness and self-esteem among students: A systematic review and meta-analysis
Source: PLoS One. 2025 Sep 24;20(9):e0329466. doi: 10.1371/journal.pone.0329466 (PMC12459768; doi:10.1371/journal.pone.0329466)
Supplement: S3 File — (DOCX) [file pone.0329466.s003.docx]

| **1.The name of the data extraction program** | No |
| --- | --- |
| **2.The data extraction schedule** | 2024.1-4 |

**2.** **All the data extracted from each study：**

|  |  |  | |  | |  |
| --- | --- | --- | --- | --- | --- | --- |
| FOMO | | | | | | |
| Servidio | 2024 | | 0.22 | | 256 | |
| Varchetta | 2023 | | 0.475 | | 589 | |
| Chi | 2022 | | 0.456 | | 938 | |
| Kostic | 2022 | | 0.34 | | 557 | |
| Fang | 2020 | | 0.45 | | 501 | |
| Shen | 2020 | | 0.43 | | 399 | |
| Fabris | 2020 | | 0.48 | | 472 | |
| Bleomen | 2020 | | 0.303 | | 831 | |
| Yin | 2019 | | 0.41 | | 704 | |
| Oberst | 2017 | | 0.461 | | 1468 | |
|  |  | |  | | 6715 | |
|  |  | |  | |  | |
|  |  | |  | |  | |
| self-esteem | | | | | | |
| Servidio | 2024 | | -0.21 | | 256 | |
| Akbari | 2023 | | -0.31 | | 3375 | |
| Ciacchini | 2023 | | -0.3 | | 258 | |
| Ahmed | 2021 | | -0.24 | | 363 | |
| Wang | 2021 | | -0.13 | | 688 | |
| Dadiotis | 2021 | | -0.18 | | 325 | |
| Acar | 2020 | | -0.29 | | 221 | |
| Pilar | 2019 | | -0.23 | | 278 | |
| Kırcaburun1 | 2018 | | -0.15 | | 804 | |
| Kırcaburun2 | 2018 | | -0.11 | | 760 | |
| Hawi | 2016 | | -0.231 | | 364 | |
|  |  | |  | |  | |
|  |  | |  | |  | |
|  |  | |  | |  | |
| loneliness | | | | | | |
| Fekih | 2023 | | 0.16 | | 363 | |
| Akbari | 2023 | | 0.34 | | 3375 | |
| Lou | 2022 | | 0.202 | | 487 | |
| Gong | 2022 | | 0.227 | | 1067 | |
| Uyaroğlu | 2021 | | 0.196 | | 555 | |
| Dadiotis | 2021 | | 0.12 | | 325 | |
| Kılınçel | 2021 | | 0.093 | | 1142 | |
| Pilar | 2019 | | 0.325 | | 278 | |
|  |  | |  | |  | |
|  |  | |  | |  | |
|  |  | |  | |  | |
| anxiety | | | | | | |
| Xiao | 2022 | | 0.32 | | 1022 | |
| Al-Mamun | 2022 | | 0.46 | | 601 | |
| Błachnio | 2021 | | 0.2 | | 1396 | |
| Kılınçel | 2021 | | 0.417 | | 1142 | |
| Sha | 2021 | | 0.33 | | 3036 | |
| Dadiotis | 2021 | | 0.21 | | 325 | |
| Blasco | 2020 | | 0.232 | | 361 | |
| Pontes | 2017 | | 0.31 | | 509 | |
| Koc | 2013 | | 0.23 | | 447 | |
|  |  | |  | |  | |
|  |  | |  | |  | |
| depression | | | | | | |
| Al-Mamun | 2022 | | 0.411 | | 601 | |
| Gong | 2022 | | 0.347 | | 1067 | |
| Xiao | 2022 | | 0.32 | | 1022 | |
| Sha | 2021 | | 0.27 | | 3036 | |
| Dadiotis | 2021 | | 0.26 | | 325 | |
| Worsley | 2018 | | 0.27 | | 1029 | |
| Kırcaburun1 | 2018 | | 0.37 | | 804 | |
| Kırcaburun2 | 2018 | | 0.22 | | 760 | |
| Pontes | 2017 | | 0.33 | | 509 | |
| Koc | 2013 | | 0.28 | | 447 | |
|  |  |  | |  | |  |

| **Author** | **Year** | **Country** | **Male/Female** | **Age(mean±SD)** | **SMA measurement** | **Education level(n)** | **Measurement instrument(Pearson's r)** | | | | |
| --- | --- | --- | --- | --- | --- | --- | --- | --- | --- | --- | --- |
|  |  |  |  |  |  |  | **Self-esteem** | **Loneliness** | **FoMO** | **Depression** | **Anxiety** |
| Servidio | 2024 | Italy | 66/190 | 23.05±3.58 | BSMAS | University | RSES | N/A | FoMOS | N/A | N/A |
| Akbari | 2023 | Iran | 1112/2263 | 15.46±1.63 | BSMAS | High school | RSES | UCLA-LS | N/A | DASS-21 | DASS-21 |
| Fekih | 2023 | Lebanon | 139/224 | 22.65±3.48 | SMDS | University | N/A | DJGLS | N/A | N/A | N/A |
| Ciacchini | 2023 | Italy | 109/149 | 17.42±1.73 | BSMAS | High school | RSES | N/A | N/A | N/A | N/A |
| Varchetta | 2023 | Span | 118/471 | 21.56±2.73 | BSMAS | University | N/A | N/A | FoMOS | N/A | N/A |
| Al-Mamun | 2022 | Bangladesh | 344/257 | 16.01±5.71 | BAFS | High school(394), Medical college(178) , University(29) | N/A | N/A | N/A | GAD-2 | PHQ-2 |
| Chi | 2022 | China | 454/484 | N/A | BSMAS | University | N/A | N/A | FoMOS | N/A | N/A |
| Fabris | 2022 | Italian | 236/236 | 13.50±1.87 | BSMAS | Middle school | N/A | N/A | FoMOS | N/A | N/A |
| Xiao | 2022 | China | 526/496 | 15.12±1.51 | CSMAS | High school | N/A | N/A | N/A | GAD-7 | CED-S |
| Kostic | 2022 | Serbia | 211/346 | 18.09±0.28 | MTUAS | High school | N/A | N/A | FoMOS | N/A/ | N/A |
| Luo | 2022 | China | 282/205 | 18.19± 0.829 | CSMSM-  DS | University | N/A | UCLA-LS | N/A | N/A | N/A |
| Błachnio | 2021 | Poland | 405/991 | 21.25±4.56 | FIS | High school(494), University(902) | N/A | N/A | N/A | N/A | GHQ-28 |
| Gong | 2021 | China | 447/620 | >18 | SNSAS-8 | University | N/A | UCLA-LS | N/A | PHQ-9 | N/A |
| KılınçelK | 2021 | Turkey | 420/722 | 15.6±2.8 | SMDS | High school | N/A | UCLA-LS | N/A | N/A | STAI |
| Sha | 2021 | China | 1305/1731 | 16.56±0.62 | SAS-SV | High school | N/A | N/A | N/A | DASS-21 | DASS-21 |
| Uyaroğlu | 2021 | Turkey | 84/471 | 30.68±11.45 | SMAS | University | N/A | SELSA‐S | N/A | N/A | N/A |
| Wang | 2021 | China | 320/368 | 13.44±0.99 | FIQ | Middle school | RSES | N/A | N/A | N/A | N/A |
| Dadiotis | 2021 | Greece | 59/266 | 21.6±5.26 | BSMAS | University | RSES | UCLA-LS | N/A | DASS-21 | DASS-21 |
| Ahmed | 2021 | Bangladesh | 167 /196 | 20.87±1.81 | BSMAS | University | RSES | N/A | N/A | N/A | N/A |
| Acar | 2020 | Turkey | 112/109 | 15.86±0.91 | SMAS | High school | RSES | N/A | N/A | N/A | N/A |
| Fang | 2020 | China | 147/354 | 19.6±1.24 | FIQ | University | N/A | N/A | FoMOS | N/A | N/A |
| Blasco | 2020 | Span | 45/316 | 22.38±10.43 | SNAQ | University(321), Master(33), Doctorate(7) | N/A | N/A | N/A | N/A | BAI |
| Shen | 2020 | China | 173/226 | 20.40±1.35 | SMAQ | University | N/A | N/A | FoMOS | N/A | N/A |
| Bloemen | 2020 | Belgium | 533/298 | 15.94±1.24 | N/A | High school | N/A | N/A | FoMOS | N/A | N/A |
| Pilar | 2019 | Span | 106/172 | 19.4±0.16 | SNAQ | University | RSES | UCLA-LS | N/A | N/A | N/A |
| Yin | 2019 | China | 301/403 | 16.8±0.92 | FIQ | High school | N/A | N/A | FoMOS | N/A | N/A |
| Worsley | 2018 | England | 259/770 | 19.80±1.67 | BSMAS | University | N/A | N/A | N/A | PHQ-9 | N/A |
| Kırcaburun | 2018 | Turkey | ①418/386 | 16.2±1.03 | SMUQ | High school（804） | RSES | N/A | N/A | SDHS | N/A |
|  |  |  | ②304/456 | 21.48±3.73 |  | University（760) |  |  |  |  |  |
| Oberst | 2017 | Spain | 377/1091 | 16.59±0.62 | SNI | High school | N/A | N/A | FoMO | N/A | N/A |
| Pontes | 2017 | Portugal | 265/244 | 13.02±1.64 | BFAS | Middle school | N/A | N/A | N/A | DASS-21 | DASS-21 |
| Hawi | 2016 | Lebanon | 190/174 | 21.1±2.3 | SMAS | University | RSES | N/A | N/A | N/A | N/A |
| Koc | 2013 | Turkey | 347/100 | 21.64±1.94 | FIQ | University | N/A | N/A | N/A | GHQ-28 | GHQ-28 |

### Note: BAFS, The Bergen Facebook Addiction Scale; GAD-2, The two-item Generalized Anxiety Disorder scale; PHQ-2, The two-item Patient Health Questionnaire; BSMAS, The Bergen Social Media Addiction Scale; FoMO, Fear of Missing Out scale; CSMAS ,The Chinese Social Media Addiction Scale; GAD-7,Anxiety symptom intensity was measured using the 7-item Generalized Anxiety Disorder Test; CES-D, Depressive symptoms were assessed using the validated Center for Epidemiological Studies Depression Scale; MTUAS, the Media and Technology Usage and Attitudes Scale; CSMSMDS, Mobile social media dependence was measured by College Students' Mobile Social Media Dependence Scale; UCLA-LA, University of California, Los Angeles-Loneliness Scale; SELSA, The Social and Emotional Loneliness Scale for Adults; DJGLS, De Jong Gierveld Loneliness Scale; FIQ, the Facebook Intrusion Questionnaire; GHQ-28, The General Health Questionnaire; SNSAS, The Chinese Social Networking Sites Addiction Scale; PHQ-9,The nine-item Patient Health Questionnaire; SMDS, Social Media Disorder scale; SAS-SV, The Smartphone Addiction Scale, Short Version; DASS-21, The Depression Anxiety Stress Scales 21; RSES, The Rosenberg Self-esteem Scale; SMAS, the Social Media Addiction Scale; SNAQ, the Social Network Addiction questionnaire; BAI, the Beck Anxiety Inventory; SMUQ, Social Media Use Questionnaire; SNI, Social network intensity scale.

**4.Confirm that the study meets the criteria for inclusion in the review：**

**Quality assessment of the included 32 studies in the meta-analysis.**

|  | Item1 | Item2 |  | Item3 | Item4 | Item5 | Item6 | Item7 | Item8 |  | Item9 | TOTAL |
| --- | --- | --- | --- | --- | --- | --- | --- | --- | --- | --- | --- | --- |
| Servidio, 2024, Italy | Y | Y |  | Y | N | Y | Y | Y | Y |  | Y | 8 |
| Akbari, 2023, Iran | Y | Y |  | Y | N | Y | Y | Y | Y |  | Y | 8 |
| Fekih, 2023, Lebanon | Y | Y |  | Y | N | Y | Y | Y | Y |  | N | 7 |
| Ciacchini, 2023, Italy | Y | Y |  | Y | N | Y | Y | N | Y |  | N | 6 |
| Varchetta, 2023, Span | Y | Y |  | Y | Y | Y | Y | Y | Y |  | N | 8 |
| AlMamun, 2020, Bangladesh | Y | Y |  | Y | Y | Y | Y | Y | Y |  | N | 8 |
| Chi, 2022, China | Y | Y |  | Y | N | Y | Y | Y | Y |  | Y | 8 |
| Fabras, 2022, Italian | Y | Y |  | Y | N | Y | Y | Y | Y |  | N | 7 |
| Xiao, 2022, China | Y | Y |  | Y | N | Y | Y | Y | Y |  | Y | 8 |
| Kostic, 2022, Serbia | Y | Y |  | Y | N | Y | Y | Y | Y |  | N | 7 |
| Luo, 2022, China | Y | Y |  | N | Y | Y | N | Y | Y |  | Y | 7 |
| Błachnio, 2021, Poland | Y | Y |  | Y | N | Y | N | Y | Y |  | Y | 7 |
| Gong, 2021, China | Y | Y |  | Y | N | Y | Y | Y | Y |  | N | 7 |
| Kılınçel, 2021, Turkey | Y | Y |  | Y | Y | N | Y | Y | Y |  | N | 7 |
| Sha, 2021, China | Y | Y |  | Y | N | Y | N | Y | Y |  | Y | 7 |
| Uyaroğlu, 2021, Turkey | N | Y |  | Y | Y | Y | Y | Y | Y |  | N | 7 |
| Wang, 2021, China | Y | Y |  | Y | N | Y | Y | Y | Y |  | Y | 8 |
| Dadiotis, 2021, Athens | Y | Y |  | N | N | Y | Y | Y | Y |  | N | 7 |
| Ahmed, 2021, Bangladesh | Y | Y |  | N | N | Y | Y | Y | Y |  | Y | 7 |
| Acar.i, 2020, Turkey | Y | Y |  | N | N | Y | Y | Y | Y |  | N | 6 |
| Fang, 2020, China | Y | Y |  | Y | N | Y | Y | Y | Y |  | N | 7 |
| Blasco, 2020, Spanish | Y | Y |  | N | N | Y | Y | Y | Y |  | Y | 7 |
| Shen, 2020, China | Y | Y |  | N | N | Y | Y | Y | Y |  | N | 6 |
| Bloemen, 2020, Belgium | Y | Y |  | Y | Y | N | N | Y | Y |  | Y | 7 |
| Pilar, 2019, Spanish | Y | Y |  | N | Y | Y | Y | Y | Y |  | N | 7 |
| Yin, 2019, China | Y | Y |  | Y | N | Y | Y | Y | Y |  | N | 7 |
| Worsley, 2018, England | Y | Y |  | Y | N | Y | Y | N | Y |  | Y | 7 |
| Kırcaburun, 2018, Turkey | Y | Y |  | Y | N | Y | Y | Y | Y |  | N | 7 |
