# Supplementary material for: Correlations between social media addiction and anxiety, depression, FoMO, loneliness and self-esteem among students: A systematic review and meta-analysis
Source: PLoS One. 2025 Sep 24;20(9):e0329466. doi: 10.1371/journal.pone.0329466 (PMC12459768; doi:10.1371/journal.pone.0329466)
Supplement: S4 File — (DOCX) [file pone.0329466.s004.docx]

**The value used to build the chart:**

|  | |  |  | |  | | |  |  |  |
| --- | --- | --- | --- | --- | --- | --- | --- | --- | --- | --- |
| FOMO | | | | | | | | | |  |
| Servidio | | 2024 | | 0.22 | | | 256 | | |  |
| Varchetta | | 2023 | | 0.475 | | | 589 | | |  |
| Chi | | 2022 | | 0.456 | | | 938 | | |  |
| Kostic | | 2022 | | 0.34 | | | 557 | | |  |
| Fang | | 2020 | | 0.45 | | | 501 | | |  |
| Shen | | 2020 | | 0.43 | | | 399 | | |  |
| Fabris | | 2020 | | 0.48 | | | 472 | | |  |
| Bleomen | | 2020 | | 0.303 | | | 831 | | |  |
| Yin | | 2019 | | 0.41 | | | 704 | | |  |
| Oberst | | 2017 | | 0.461 | | | 1468 | | |  |
|  | |  | |  | | | 6715 | | |  |
|  | |  | |  | | |  | | |  |
|  | |  | |  | | |  | | |  |
| self-esteem | | | | | | | | | |  |
| Servidio | | 2024 | | -0.21 | | | 256 | | |  |
| Akbari | | 2023 | | -0.31 | | | 3375 | | |  |
| Ciacchini | | 2023 | | -0.3 | | | 258 | | |  |
| Ahmed | | 2021 | | -0.24 | | | 363 | | |  |
| Wang | | 2021 | | -0.13 | | | 688 | | |  |
| Dadiotis | | 2021 | | -0.18 | | | 325 | | |  |
| Acar | | 2020 | | -0.29 | | | 221 | | |  |
| Pilar | | 2019 | | -0.23 | | | 278 | | |  |
| Kırcaburun1 | | 2018 | | -0.15 | | | 804 | | |  |
| Kırcaburun2 | | 2018 | | -0.11 | | | 760 | | |  |
| Hawi | | 2016 | | -0.231 | | | 364 | | |  |
|  | |  | |  | | |  | | |  |
|  | |  | |  | | |  | | |  |
|  | |  | |  | | |  | | |  |
| loneliness | | | | | | | | | |  |
| Fekih | | 2023 | | 0.16 | | | 363 | | |  |
| Akbari | | 2023 | | 0.34 | | | 3375 | | |  |
| Lou | | 2022 | | 0.202 | | | 487 | | |  |
| Gong | | 2022 | | 0.227 | | | 1067 | | |  |
| Uyaroğlu | | 2021 | | 0.196 | | | 555 | | |  |
| Dadiotis | | 2021 | | 0.12 | | | 325 | | |  |
| Kılınçel | | 2021 | | 0.093 | | | 1142 | | |  |
| Pilar | | 2019 | | 0.325 | | | 278 | | |  |
|  | |  | |  | | |  | | |  |
|  | |  | |  | | |  | | |  |
|  | |  | |  | | |  | | |  |
| anxiety | | | | | | | | | |  |
| Xiao | | 2022 | | 0.32 | | | 1022 | | |  |
| Al-Mamun | | 2022 | | 0.46 | | | 601 | | |  |
| Błachnio | | 2021 | | 0.2 | | | 1396 | | |  |
| Kılınçel | | 2021 | | 0.417 | | | 1142 | | |  |
| Sha | | 2021 | | 0.33 | | | 3036 | | |  |
| Dadiotis | | 2021 | | 0.21 | | | 325 | | |  |
| Blasco | | 2020 | | 0.232 | | | 361 | | |  |
| Pontes | | 2017 | | 0.31 | | | 509 | | |  |
| Koc | | 2013 | | 0.23 | | | 447 | | |  |
|  | |  | |  | | |  | | |  |
|  | |  | |  | | |  | | |  |
| depression | | | | | | | | | |  |
| Al-Mamun | | 2022 | | 0.411 | | | 601 | | |  |
| Gong | | 2022 | | 0.347 | | | 1067 | | |  |
| Xiao | | 2022 | | 0.32 | | | 1022 | | |  |
| Sha | | 2021 | | 0.27 | | | 3036 | | |  |
| Dadiotis | | 2021 | | 0.26 | | | 325 | | |  |
| Worsley | | 2018 | | 0.27 | | | 1029 | | |  |
| Kırcaburun1 | | 2018 | | 0.37 | | | 804 | | |  |
| Kırcaburun2 | | 2018 | | 0.22 | | | 760 | | |  |
| Pontes | | 2017 | | 0.33 | | | 509 | | |  |
| Koc | | 2013 | | 0.28 | | | 447 | | |  |
|  | |  |  | |  | | |  |  |  |
| **Points extracted from the image for analysis/ Values behind reported means, standard deviations, and other measures:** | | | | | | | | | | |
| Factors | | Summary r | | | 95%CI | | I^2^ | | | P |
| Anxiety | | 0.31 | | | (0.25, 0.36) | | 87.90% | | | <0.001 |
| Depression | | 0.31 | | | (0.27, 0.34) | | 69.40% | | | <0.001 |
| Loneliness | | 0.21 | | | (0.13, 0.29) | | 90.90% | | | <0.001 |
| FoMO | | 0.41 | | | (0.36, 0.45) | | 79.80% | | | <0.001 |
| Self-esteem | | -0.24 | | | (-0.26, -0.22) | | 80.90% | | | <0.001 |
|  | |  | | |  | |  | | |  |
