# Supplementary material for: Correlations between social media addiction and anxiety, depression, FoMO, loneliness and self-esteem among students: A systematic review and meta-analysis
Source: PLoS One. 2025 Sep 24;20(9):e0329466. doi: 10.1371/journal.pone.0329466 (PMC12459768; doi:10.1371/journal.pone.0329466)
Supplement: S6 Appendix — (DOCX) [file pone.0329466.s006.docx]

**APPPENDIX A** JBI critical appraisal checklist for studies reporting prevalence data.

|  | Yes | No | Unclear | Not applicable |
| --- | --- | --- | --- | --- |
| 1. Was the sample frame appropriate to address the target population? |  |  |  |  |
| 2. Were study participants sampled in an appropriate way? |  |  |  |  |
| 3. Was the sample size adequate? |  |  |  |  |
| 4. Were the study subjects and the setting described in detail? |  |  |  |  |
| 5. Was the data analysis conducted with sufficient coverage of the identified sample? |  |  |  |  |
| 6. Were valid methods used for the  identification of the condition? |  |  |  |  |
| 7. Was the condition measured in a  standard, reliable way for all participants? |  |  |  |  |
| 8. Was there appropriate statistical analysis? |  |  |  |  |
| 9. Was the response rate adequate, and if not, was the low response rate managed appropriately? |  |  |  |  |

Quality assessment adapted from: Munn Z, Moola S, Lisy K, Riitano D, Tufanaru C. Methodological guidance for systematic reviews of observational epidemiological studies reporting prevalence and incidence data. Int J Evid Based Healthc. 2015;13(3):147–153.
