# Supplementary material for: Correlations between social media addiction and anxiety, depression, FoMO, loneliness and self-esteem among students: A systematic review and meta-analysis
Source: PLoS One. 2025 Sep 24;20(9):e0329466. doi: 10.1371/journal.pone.0329466 (PMC12459768; doi:10.1371/journal.pone.0329466)
Supplement: Attachment — Submitted filename: To reviwer 1.docx [file pone.0329466.s008.docx]

Dear Reviewer 1,

Thank you for your careful consideration of our manuscript “Correlations between social media addiction and anxiety, depression, FoMO, loneliness and self-esteem among students: A systematic review and meta-analysis”. We are grateful for the constructive feedback provided by the reviewers, which we believe has significantly improved the quality and clarity of our work. The black font is your suggestion, while the blue font is the change we made based on your suggestion

The title:

1. The title is clearly formulated and unambiguous, effectively conveying the focus of the study.

The abstract:

1. The abstract clearly outlines the research problem, research methodology, research processes followed in the study, relevant findings, and the implications of the study.

2. There are no page numbers in this manuscript, which may make it difficult to highlight areas for potential revision. Please consider page 1 as where the abstract is located, with page 22 marking the beginning of the reference list. Suggestions for the abstract are:

Reply: We appreciate the reviewers' detailed and helpful suggestions on the abstract. In the lower left corner of the article, we marked the page number. According to your comments, we have made modifications which are displayed in Table 1. Thank you again for your positive comments and valuable suggestions to improve the quality of our manuscript.

Table 1: A description of modifications to the abstract section

| a. Page 1, line 16: Avoid using the phrase “and so on” as it lacks academic tone. | Page 1, Line 26: We have delete “and so on” and supplemented with specific social networking platforms (SNS). |
| --- | --- |
| b. Page 1, line 16: Please clarify what “It” refers to. Does “it” refer to the Internet, social media usage or social media addiction? | Page 1, Line 26: It refer to the social media addiction. We have used the social media addiction to replace the “it”. |
| c. Page 1, line 16: Replace “tight links” with more academic language such as “significant associations”. | Page 1, Line 27: We have used “significant associations” replace the “tight links”. |
| d. Page 1, line 16: Please remove the “the” before psychological issues. | Page 1, Line 27: We have removed “the” before mental health concerns |
| e. Page 1, line 17: Consider using “mental health concerns” instead of “psychological issues”. | Page 1, Line 27: We have used “mental health concerns” instead of “psychological issues” |
| f. Page 1, line 18: First mention social media addiction, then follow with the abbreviation (SMA). | Page 1, Line 26: We have written “social media addiction (SMA)” when SMA first appear. |
| g. Page 1, line 19: Please clarify what “these factors” refer to. | Page 1, Lines 31-32：We have clarified these factors (anxiety, depression, self-esteem, FoMO and loneliness) the section of abstract. |
| h. Page 1: Consider explaining why the combination of anxiety, depression, loneliness, self-esteem, and FOMO was investigated. This should also be clarified in the rationale and discussion sections. | Page 1, Lines 26-29: We wish to investigate the reasons that contribute to social media addiction. After examining the literature, we found that these factors highly occurred, so we decided to choose these factors to do a comprehensive analysis. We also briefly explain the reasons on Page 1, Lines 26-29. |
| i. Page 2, lines 9 – 13: Please rephrase these sentences for clarity, as the arguments currently do not make sense. Language editing may help here. | Page 2, Lines 54-58: We have rephrased these sentences to make the text more clearly and meaningful. |

**Introduction:**

1. The introduction/rationale section was approached with interest. Thank you.

2. The rationale section presents valid arguments and the importance of the study is clearly indicated and well-argued.

3. Please ensure that important arguments are supported by citations. Please see:

Reply: We sincerely appreciate the valuable comments. We have checked the literature carefully and added more reference into the revised manuscript. In order to save your effort, we have filled the table with the references you proposed that need to be added to facilitate your review. The serial number is the order of the references in the article. In addition to the supplementary literature in the table, we also supplemented the literature on low self-esteem and social media addiction (25) in Page 4, Line 108.

Table2: Supplementary notes to references

| a. Page 2, line 22. | Page 3, Line 67: (2) |
| --- | --- |
| b. Page 2, line 28. | Page 3, Line 73: (4) |
| c. Page 2, line 29. | Page 3, Line 75: (5) |
| d. Page 3, line 7. | Page3 Lines 79-83: We have supplemented the literature on the development of SMA as an important academic issue (11) and introduced specific data in recent years to support it. (10) |
| e. Page 4, line 4. | Page 5, Line 111: (27) |
| f. Page 17, line 27 | Page 24, Line 342: (77) |
| g. Page 18, line 6. | Page 25, Lines 364, 366: (80) (81) |

4. Page 3: An argument is made that the number of social media addicts is increasing. What studies or statistics support this argument? The authors have not provided any statistics or references to substantiate this important argument.

Reply: We are appreciative of the reviewers' insightful comments, which has strengthened the article's validity. We completely agree with this recommends, and to support this essential point, and added the statistic to verify the increasing of SMA addicts in Page 3, Lines 80-81.

5. Page 3, line 7: Please avoid the phrase “hot topic”, as it lacks academic tone. Instead, refer to it as “a subject of significant interest”.

Reply: We have revised the “hot topic” to “a subject of significant interest” in Page3 Line 82.

6. Page 3, line 12: The phrase “are fewer opportunities” should perhaps be “there were fewer opportunities,” as the lockdown is now over

Reply: We have revised the “are fewer opportunities” to “there were fewer opportunities,” in Page 3, Line 88.

However, due to the irrelevance of the novel coronavirus to this paper, this supplementary content has been deleted.

7. Is social media addiction a global concern regardless of COVID-19? The rationale has a strong focus on the pandemic period, but it would be beneficial to highlight whether SMA is a global concern outside of the COVID-19 context. It is unclear whether the authors intend to focus solely on the COVID-19 period.

Reply: We would like to extend our sincere gratitude for your thoughtful consideration of our manuscript and the constructive feedback provided. Our study was a meta-analysis conducted outside the context of the pandemic, and the data in all the articles collected were not limited to the COVID-19 period. We agree that mentioning the COVID-19 influence in the introduction was not pertinent, and thus, we have removed all such references to ensure clarity and relevance (in Pages 3-4, Lines 84-94; Page 5, Lines 116-118, 123-126). We greatly appreciate your expert review and the invaluable suggestions you have offered. Your feedback has been crucial in refining our work.

8. Page 4, lines 17 to 18: Please rephrase for better clarity.

Reply: Thank for your precious comment, we have rephrased the sentence to “Since the COVID-19 pandemic has been raging, people spend a lot of time on social media for information focus on updates on the pandemic, prevention methods and effective treatment.”

Upon careful consideration, it has been determined that the content related to the novel coronavirus is not directly pertinent to the scope and focus of this manuscript. Consequently, the supplementary material addressing this topic has been deemed inessential and has been removed to maintain the relevance and coherence of the paper.

9. Page 4, line 22: Why is the emphasis only on developing effective interventions for teenagers? Many studies indicate that university/college students are the highest users of social media. Why not also advocate for effective interventions for these students?

Reply: The selection of the term “teenagers” was our negligence. What we meant was the student group. Moreover, the intervention measures are beyond the research scope of this article. More experiments should be carried out to explore the correct intervention methods. Therefore, we are decided to delete this paragraph in Page 5, Lines 128-129,131.

10. Page 3, line 20: Consider using “mental health issues” rather than “mental issues”.

Reply: Thank you for pointing out the shortcomings in the use of language in the article. We have revised “mental issues” into “mental health issues” in Page 4, Lines 96-97.

11. The rationale for the combination of anxiety, depression, loneliness, self-esteem, and FOMO remains unclear after reading this section.

Reply: The reason for combining these factors in this paper is that it is found that these symptoms are relatively common in patients with social media addiction when consulting data. Moreover, according to other experiments conducted by our research group, these symptoms are also relatively common in clinical practice in patients with Internet addiction. We will summarize the specific reasons in the DISCUSSION part in Page 23, Lines 323-330)

12. Strengthen the rationale section by incorporating more literature to support the arguments presented.

Reply: Thank for your professional and detailed suggestions, we have learned that our article needs more relevant literature support, so we have supplemented the corresponding references in the position you pointed (can be checked in Table 2).

Methodology:

1. The methodology section was discussed properly.

2. Page 5, line 10: Clarify what the six elements refer to. Please elaborate.

Reply: We have clarified what the factors (anxiety, depression, loneliness, self-esteem, and FOMO) refer to in Page 6, Lines 145-146.

3. Page 5, line 24 and line 27: Reference is made to self-esteem in line 24 and then again in line 27. Should self-esteem be replaced with FOMO in line 27?

Reply: We are grateful for your careful review of this article. This error is due to our carelessness, FoMO can’t replace self-esteem. We have made revisions in Page 7, Line 166.

4. The manuscript indicates that 32 studies were included in the meta-analysis. However, only 31 studies are listed in Table 1.

Reply: Thank you for your careful review. We have revised table 1 and table 2 (in Pages 9-13 and Pages 14-16). To ensure that the documents included in the meta-analysis are correctly filled in the form, and to ensure the consistency of the full text.

5. Discrepancies exist between Tables 1 and 2. In Table 1, a study is listed as Fabris (2022) – Italian, which does not appear in Table 2. In Table 2, two studies are listed as Kitiş (2022) – Turkey, and Koc (2013) – Turkey, which do not appear in Table 1. Please ensure that the correct studies are reported on.

Reply: Thank you very much for your careful review of this manuscript. We reviewed the manuscript carefully, then revised Table 1 and Table 2. We made the order of documents in Table 1 and Table 2 (in Pages 9-13 and Pages 14-16) consistent and ensured the consistency of the full text.

**Results:**

Reply: We sincerely thank your valuable feedback that we have used to improve the quality of our manuscript. For the convenience of reference, the reviewer's comments are listed in the table below and numbered. Our changes to the manuscript are given in blue text. Your comments have improved the quality of the article, and we thank you again for your review and the effort you put into it.

Table 3: A description of modifications to the results section

| 1. The presentation of the results was done in a systematic and structured manner. The results were presented using figures and tables and were discussed properly. |  |
| --- | --- |
| 2. Page 12, line 18: Rather refer to the fact that 1315 studies were excluded rather than “should not have been included”. | In Page 17, Line 212: We have used the “have been deleted” to replace the “should not have been included”. |
| 3. Page 13, line 1: Rather refer to “screening” the titles and abstracts of the sources rather than “browsing”. | In Page 18, line 214: We have used the “screening” to replace the “browsing”. |
| 4. Page 13, line 2: What does “researches” mean” | In Page 18, line 215: We have modified the “researches” to “articles”. |
| 5. Page 13, line 5: Reference is made to 27 studies whereas 32 studies are referred to in the manuscript. | In Page 18, line 218: We have changed 27 to the correct number (32 studies). |
| 6. Page 13, line 13: … nine studies that reported…. | In Page 19, line 227-232: In the results section, each subsection will provide a detailed account of the number of articles included in the meta-analysis for the associations between SMA and anxiety, depression, loneliness, and FoMO. We have deleted this  paragraphs to avoid redundancy. |
| 7. The rationale for combining anxiety, depression, loneliness, self-esteem, and FOMO remains unclear in this section as well. | In Pages 23, Lines 323-330：The reason for combining these factors in this paper is that it is found that these symptoms are relatively common in patients with social media addiction when consulting data. Moreover, according to other experiments conducted by our research group, these symptoms are also relatively common in clinical practice in patients with Internet addiction. We will summarize the specific reasons in the DISCUSSION part (in Pages 23, Lines 323-330) |
| 8. Page 16, line 13: Is the abbreviation SMA missing from the | In Page 22, line 300： SMA has been added to the title of the figure. |

Discussion:

Reply: Thank you for your helpful feedback on our article. We've made changes based on your suggestions and put them in a table for you to see easily. Your comments are important to us, and we've taken them all into account. Here's the table with your comments on the left and our changes on the right. We really appreciate your ideas to make our article better.

Thanks again for your suggestions

Table 3: A description of modifications to the discussion section

| 1.The discussion of the findings was done well. |  |
| --- | --- |
| 2. Page 16, line 22: Refer back to the earlier comment regarding the use of the term “hot topic”. | In Page 23, line 318: We have modified "hot topic" to "a subject of significant interest" according to your previous modification suggestions. |
| 3. Page 17, line 1: Use “results” instead of “result”. | In Page 24, line 332: We have used “results” instead of “result”. |
| 4. Page 17, line 1: Clarify what the authors consider to be weak correlations. For instance, a correlation of .41 could be considered moderate. | In Pages 24, Lines 332: We have changed the “weak correlation” to “weak to moderate correlation” |
| 5. Page 17, line 1: What does MPA refer to? | In Page 24, line 333: We apologize for the error in our document where "SMA" was mistakenly written as "MPA." The mistake has been corrected |
| 6. Page 17, lines 9 and 10: Should FOMO not form part of this argument? | In Page 24, line 341: We agree with your suggestion to incorporate FoMO into this argument. |
| 7. Page 17, line 20: Provide brief information on the countries where the studies were conducted, as well as the sample sizes, similar to how it was done in other sections. | In Pages 24-25, line 352-356: We have provided a concise overview of the country in which the study was conducted, along with the sample size in the part of SMA and anxiety and depression. |
| 8. Page 19, line 4: Please rephrase. | Page 26, line 379-380: In order to maintain the focus on the strength of the relationship between FoMO and the variables of interest, without drawing comparisons with other factors, we have delected to remove the sentence that contrasted FoMO's correlation with those of other elements. |
| 9. Page 19, line 17: Do you mean the RSES? | Page 27, Line 411: We sincerely apologize for the inadvertent error that resulted in the incorrect transcription of "RSES" as "RESE" due to our carelessness. We have taken immediate action to correct this mistake, and the text now accurately reflects "RSES." |
| 10. Page 21, line 2: Consider using the term depression throughout instead of terms such as sadness or despair. | Page 24, line 341 and Page 29, line 454：We've changed "sadness or despair" to "depression" |
| 11. Including more literature to support the arguments presented would enhance the overall quality of the manuscript. | As per your request, we have supplemented the article with relevant literature to enrich its content. For specifics, please refer to Table 2 in the response letter. |

Once again, we would like to express our gratitude to the reviewers for their insightful comments and to you for overseeing the review process. We hope that our revisions meet with your approval and that our manuscript can be considered for publication in your esteemed journal.

Thank you for your time and consideration.

Best regards,

Zhangjing
