# Supplementary material for: Correlations between social media addiction and anxiety, depression, FoMO, loneliness and self-esteem among students: A systematic review and meta-analysis
Source: PLoS One. 2025 Sep 24;20(9):e0329466. doi: 10.1371/journal.pone.0329466 (PMC12459768; doi:10.1371/journal.pone.0329466)
Supplement: Attachment — Submitted filename: Rebuttal letter (2).docx [file pone.0329466.s009.docx]

**To editor:**

**Dear Editor,**

**Thank you very much for sharing the peer review feedback on my manuscript titled "Correlations between Social Media Addiction and Anxiety, Depression, FoMO, Loneliness, and Self-Esteem among Students: A Systematic Review and Meta-Analysis." I truly appreciate the time and effort the editor and reviewers have dedicated to providing such valuable comments. In response to the feedback, I have carefully considered each comment and made thorough revisions to address the concerns raised.** **In order to ensure the quality of the manuscript, we have taken your suggestions to heart and had it polished on the AJE website. The polishing report has been uploaded as an attachment.**

**We would like to express our sincere gratitude to all the editors and reviewers for their valuable comments and suggestions. Below, l provide a point-by-point response to each reviewer's comments. We hope that the revision would satisfactorily address the comments and concerns of the editors and reviewers.**

**Yours sincerely,**

**Zhang jing**

**To reviewer #1:**

**Dear reviewer：**

**Thank you very much for your positive feedback. We have carefully revised the manuscript based on your comments. We hope that these revisions and improvements will satisfactorily address the professional issues you raised. Our point-to-point responses are as follows.**

• Page 5, line 113: I think the word “and” is missing in the sentence. “…have been examined more and were chosen….”

**Response：感谢你专业的意见我们已经在句子中加入and on line 124, page5**
• Page 5, line 123: Remove the word “used” as the word “using” already appears in the sentence.

**Response：We are grateful for your careful review. The word "used" has been removed on line 135, page 6.**
• Page 5, line 127: I think RSES is omitted from the sentence. Should the sentence read “…were restricted to RSES or SISE….”

**Response：We apologize for our oversight. We have added RSES to the sentence on line 140, page 6, to ensure the smoothness and coherence of the article.**
• Page 5, line 129: Replace the full stop with a semicolon for consistency.

**Response：We have replaced the period with a semicolon on line 142, page 6.**
• Page 5, line 130: Rep 6.ace the full stop with a semicolon for consistency.

**Response：We have replaced the period with a semicolon on line 144, page 6.**
• Page 15, line 180: Replace showed with shows.

**Response：we have replaced showed with shows on line 198, page 16. Thank you again for your time on this manuscript.**
• Page 20, line 283: Did you collect 8839 student participants from seven countries?

**Response：We are deeply grateful for your professional and meticulous feedback. We regret any confusion that may have arisen from our initial phrasing. It was not our intention to imply that we collected the data ourselves; instead, our focus was on analyzing the data presented in the articles. The sentence has been revised to:**

**"In our meta-analysis, we analysed the correlation between anxiety and SMA. We collected 11 articles, which included a total sample size of 8,839 students from seven countries: China, Poland, Bangladesh, Turkey, Greece, Spain, and Portugal."**

**Thank you for your meticulous review. We apologize for our oversight. In response to your comments, we have made the necessary corrections to ensure consistency and harmony throughout the manuscript.**

**Yours sincerely,**

**Zhang jing**

**To reviewer #2:**

**Dear reviewer：**

**We feel great thanks for your professional review work on our article. As you are concerned, there are several problems that need to bead dressed. According to your nice suggestions. we have made corrections to our manuscript and the detailed corrections are listed below.**
1. line 197: please carefully revise " state mindfullness" to SMA

**Response：Thanks for your careful review, we have revised it to on line 218, page 17.**
2. line 204: "significative" to significant

**Response：Thank you for your suggestion. We have revised "significative" to "significant" on line 226, page 18.**
3. in figure 2,3,4,5 caption what is abbreviated as PUSM? I think this need revision

**Response：We are thankful for your careful review. We have modified Figure 2,3,4,5 and re-uploaded it to the system.**
4. I suggest the author/s go through the document and edit for some typos, grammatical error, and coherence

**Response：Thank you for your professional review. We apologize for our careless. In response to your comments, we have made the necessary corrections to ensure consistency and harmony throughout the manuscript. Consequently, we have decided to engage the services of the professional editing website AJE to further polish our article, with the hope that it will fully meet your requirements.**

**Thank you very much for your attention and time. Look forward to hearing from you.**

**Yours sincerely,**

**Zhang jing**
