# Supplementary material for: Correlations between social media addiction and anxiety, depression, FoMO, loneliness and self-esteem among students: A systematic review and meta-analysis
Source: PLoS One. 2025 Sep 24;20(9):e0329466. doi: 10.1371/journal.pone.0329466 (PMC12459768; doi:10.1371/journal.pone.0329466)
Supplement: Attachment — Submitted filename: response4_letter.docx [file pone.0329466.s010.docx]

Dear Reviewers:

We sincerely appreciate your valuable comments. In response to your suggestions, we have carefully reviewed the manuscript and accordingly implemented the following modifications:

(1) Line 58: Introduce global data and then specify China.

We thank you for this constructive suggestion. To enhance the logical flow of the paper, we have accordingly supplemented the manuscript with the following additions, accompanied by proper citation to the data sources:

As of January 15, 2025, there were 5.40 billion internet users, equivalent to 66% of the world’s population. And the China Internet Network Information Center (CNNIC) published the 51st Statistical Report on the Development Status of the Internet in China in March 2022. (in page 2, lines 58-61)

(2) Lines 62 and 64: The phrase "On the one hand" is repeated.

We appreciate your insightful feedback. To improve textual coherence, we have rephrased the indicated passage as follows:

On the one hand, due to the large influx of information, internet users are constantly confirming the validity and trustworthiness of information on social media; on the other hand, this feature increases dependence on social media owing to the increased demand for information. (in pages 2-3, lines 63-67)

(3) Line 79: The sentences dealing with depression and self-esteem need to be better connected.

We are particularly grateful for this crucial observation. In response to your comment, we have substantially strengthened the conceptual linkage between depression and self-esteem in the revised version, with the following key enhancements:

Self-esteem is a subjective evaluation that refers to how people feel about themselves and numerous studies have shown a negative correlation between SMA and self-esteem, which may be related to the greater need for people with low self-esteem to cultivate identity through social media. (in page 3, lines 82-85)

(4) Line 86: A linking paragraph is needed to talk about loneliness. The transition is abrupt.

This perspicacious critique has brought to light critical structural inconsistencies in our original exposition. With profound gratitude for your scholarly vigilance, we have implemented revisions that fundamentally reconstitute the argumentation framework through:

Meanwhile, with the advancement of society and the deepening division of labor, loneliness has emerged as a significant contributing factor to SMA. After extensive research, researchers classified loneliness according to its causes, which can be divided into emotional loneliness and social loneliness. (in page 4, lines 89-91)

(5) Line 99: Provide social, theoretical, and methodological justification for the study. The variables of interest need to be better connected. The objectives need to be clearly presented.

We are profoundly grateful for your seminal critique regarding the conceptual scaffolding of our literature review. In direct response to your astute observations, we have conducted a refinement of the research motivation:

According to the published research, anxiety, depression, loneliness, FoMO and self-esteem are the most common factors in SMA among adolescents. This article aims to explore the relationship between SMA and anxiety, depression, loneliness, FoMO and self-esteem, in order to understand the etiology of SMA and provide new approaches for prevention or treatment. As a populous country and a developing country that is rapidly digitizing, China is confronted with a severe challenge: the sharp increase in the number of teenagers suffering from SMA has escalated into an urgent public health issue. Therefore, studying the psychological roots of this phenomenon has become an urgent priority. Based on the above purposes, this study can deepen our understanding of addictive behaviors and help prevent adverse effects on students' physical and mental health. (in pages 4-5, lines 105-115)

(6) Start by restating the objectives of the study.

We really appreciate the effort you have put into this article. In response to your professional and detailed comments, we have made improvements: Furthermore, this article can provide more comprehensive guidance for various approaches to intervention, government policy-making and the classification of addictive behavior. Enhance the public's awareness of preventing Internet addiction, improve their online media literacy and protection skills, and ensure healthy and civilized Internet use. (in page 21, lines 294-298)

(7) Present the limitations and also the possible solutions, and how they can be remedied.

We sincerely appreciate your insightful comments, which have helped us better recognize the limitations of our current study. Through careful reflection, we have identified several key areas requiring improvement in future research:

However, methodological constraints include (a) underpowered sample cohorts in the meta-analysis, and (b) ethnocentric recruitment practices in the source studies, failing to represent cross-cultural populations. We will do our best to make improvements in the future work. (in page 26, lines 403-406)

Once again, we would like to express our gratitude to the reviewers for their insightful comments and to you for overseeing the review process. We hope that our revisions meet with your approval and that our manuscript can be considered for publication in your esteemed journal.

Thank you for your time and consideration.

Best regards,

Zhangjing
